# Synergetic effect of dilute acid and alkali treatments on fractional application of rice straw

**DOI:** 10.1186/s13068-016-0632-9

**Published:** 2016-10-18

**Authors:** Shaolong Sun, Weijing Chen, Jianing Tang, Bing Wang, Xuefei Cao, Shaoni Sun, Run-Cang Sun

**Affiliations:** Beijing Key Laboratory of Lignocellulosic Chemistry, Beijing Forestry University, Beijing, 100083 China

**Keywords:** Rice straw, Dilute acid pretreatment, Alkali treatment, Value-added application

## Abstract

**Background:**

The biorefinery based on an effective and economical process is to fractionate the three primary constituents (cellulose, hemicelluloses, and lignin) from lignocellulosic biomass, in which the constituents can be respectively converted into high-value-added products. In this study, a successive treatment with dilute acid (0.25–1.0 % aqueous H_2_SO_4_, 100–150 °C, 0.5–3.0 h) and alkali (1.5 % aqueous NaOH, 80 °C, 3 h) was performed to produce xylooligosaccharides (XOS), high-purity lignin, and cellulose-rich substrates to produce glucose for ethanol production from rice straw (RS).

**Results:**

During the dilute acid pretreatment, the maximum production of XOS (12.8 g XOS/100 g RS) with a relatively low level of byproducts was achieved at a relatively low temperature (130 °C) and a low H_2_SO_4_ concentration (0.5 %) for a reaction time of 2.0 h. During the alkali post-treatment, 14.2 g lignin with a higher purity of 99.2 % and 30.3 g glucose with a higher conversion rate by enzymatic hydrolysis were obtained from the successively treated substrates with 100 g RS as starting material. As the pretreatment temperature, H_2_SO_4_ concentration, or time increased, more *β*-*O*-4 linkages in lignins were cleaved, which resulted in an increase of phenolic OH groups in lignin macromolecules. The signal intensities of G_2_ and G_6_ in HSQC spectra gradually reduced and vanished, indicating that a condensation reaction probably occurred at C-2 and C-6 of guaiacyl with the side chains of other lignin.

**Conclusions:**

The present study demonstrated that the successive treatments with dilute acid and alkali had a synergetic effect on the fractionation of the three main constituents in RS. It is believed that the results obtained will enhance the availability of the combined techniques in the lignocellulosic biorefinery for the application of the main components, cellulose, hemicelluloses, and lignin as biochemical and biofuels.

**Electronic supplementary material:**

The online version of this article (doi:10.1186/s13068-016-0632-9) contains supplementary material, which is available to authorized users.

## Background

A significant type of biomass burning is a common way to eliminate waste after harvesting in China, such as in Shandong and Hebei Provinces, which is an important source of greenhouse gases and particulate pollutants in the atmosphere and has a remarkable effect on global atmospheric chemistry and global warming [1, 2]. To avoid this pollution, many new schemes, including papermaking and bioethanol production are developed for the application of straw [3, 4]. These schemes mainly focus on the value-added application of cellulose. Note that rice straw consists of three major biomacromolecules, namely cellulose, hemicelluloses, and lignin, and each of these components has a distinct and complex structure [5, 6]. Thus, rice straw can be considered as a potential renewable source of energy and biobased chemicals for economic development and environmental sustainability [7]. Unfortunately, although cellulose has been extensively applied in many fields, such as paper, food, and plastics industries as the predominant component of biomass, other components have not been fully utilized, particularly lignin [8, 9].

The ultimate goal of rice straw separation according to the biorefinery is to obtain the fractionation of the three primary constituents via an effective and economical process, in which the three fractions can be converted into multiple biobased products, respectively. To date, a series of biorefinery processes for biomass including dilute acid, alkali, and steam explosion treatments have been investigated [10–12]. Among them, dilute acid pretreatment causes structural changes of lignin and cellulose as well as degradation of hemicelluloses, which in turn contribute to the reduction of biomass recalcitrance [13]. Simultaneously, xylooligosaccharides (XOS) were produced from hemicelluloses in biomass during the dilute acid pretreatment. More importantly, the pretreatment accelerates the subsequent delignification [5]. As a biorefinery process, there is a growing interest in lignin, the third most abundant component in biomass, for developing biobased materials and chemicals. In a typical cellulosic biomass, lignin fills the spaces between cellulose and hemicelluloses, holding the biomass matrix together [14]. Hence, it is necessary to isolate lignin from the pretreated biomass and to understand the structural features, which determine the final utilization of lignin. For example, lignin can be used as a substite for phenol in producing phenol–formaldehyde resins [15]. Interestingly, alkali treatment is a very effective method to separate lignin, especially from the pretreated biomass. It can further disrupt the cell wall by partially dissolving lignin and then obtain cellulose-rich residues [16]. Cellulose-rich residues can be further converted to glucose with high yields by enzymatic hydrolysis, which provides a high efficient way for bioethanol production.

As far as we know, although both acid and alkali pretreatments for the enzymatic hydrolysis of biomass have been intensively studied [17–20], very little concern is given to the fractional applications of the main components, cellulose, hemicelluloses, and lignin as biochemical and biofuels, especially during a combined treatment based on successive dilute acid and alkali treatments. For instance, in order to achieve high enzymatic hydrolysis efficiency, corncob was successively pretreated with acid and alkali to remove the non-cellulosic components [17]. Similarly, Kim et al. [18] reported the effects of sequential acid/alkali pretreatment on the enzymatic digestibility enhancement of empty palm fruit bunch fiber. For rice straw, sole dilute sulfuric acid or sole dilute alkali or combinations of them (dilute sulfuric acid and aqueous ammonia) have been studied [21–23]. However, these studies only focus on improving the enzymatic hydrolysis efficiency of the pretreated biomass. The recovery of hemicelluloses and lignin has not been investigated. Additionally, in almost all the cellulosic ethanol technologies, in pre-commercial stage, most of the hemicelluloses are preserved during pretreatment and then fermented into ethanol, while the structure and further utilization of the residual lignin are rarely reported.

In this study, a combination of successive dilute acid (0.25–1.0 % aqueous H_2_SO_4_, 100–150 °C, 0.5–3.0 h) and alkali (1.5 % aqueous NaOH, 80 °C, 3 h) treatments was proposed to enhance availability for the application of three major components (cellulose, hemicelluloses, and lignin) as biochemical and biofuels (XOS, high-purity lignin, and cellulose-rich substrates to produce glucose for ethanol production). The composition of XOS was determined by a high-performance anion-exchange chromatography (HPAEC). The yield, purity, molecular weight, and structural transformation of alkaline lignin isolated have been thoroughly investigated. Meanwhile, the cellulose-rich substrates were characterized by chemical constituent, scanning electron microscopy (SEM), Fourier transform infrared (FT-IR), and X-ray diffraction (XRD), and the yield of glucose was also determined. These results will provide useful information in the utilization of rice straw for the value-added biochemicals or bioethanol in biorefinery industry.

## Results and discussion

### Effects of successive dilute acid and alkali treatments on the chemical constituent, surface morphology, and crystallinity of the substrate

**Fig. 1:**
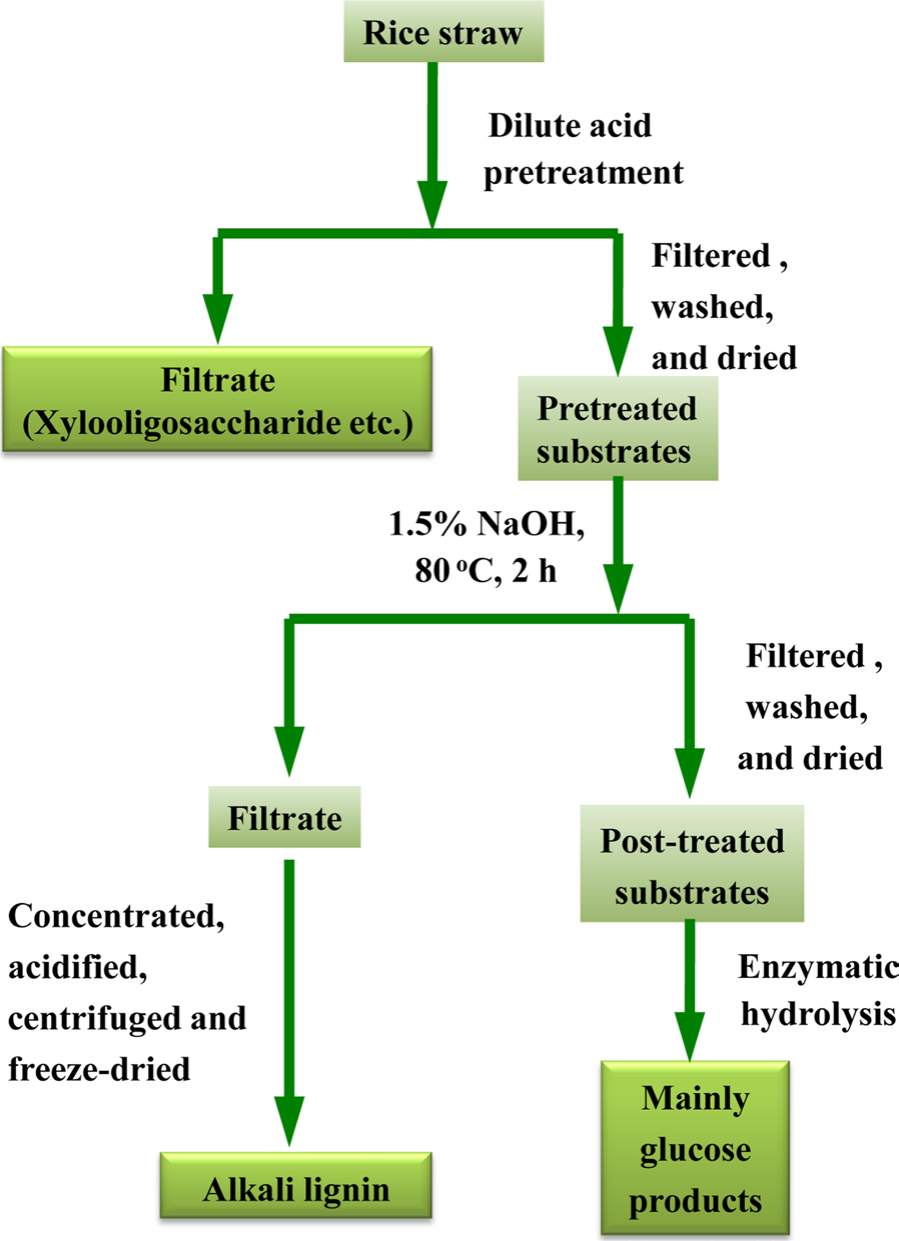
Schematic illustration of the combined biorefinery processes

A biorefinery with successive dilute acid and alkali treatments was applied to enhance the value-added application of RS in this study. The scheme of the biorefinery process is shown in Fig. 1, and the corresponding solid yields obtained at various pretreating conditions are shown in Table 1. As shown, the solid yields decreased from 76.54 to 56.15/100 g RS as the pretreatment temperature, H_2_SO_4_ concentration, or time increased. This is mainly due to the degradation of hemicelluloses, which was further confirmed by the chemical constituents and FT-IR spectral analysis of the dilute acid pretreated substrates (Table 2; Additional file 1: Figure S1). During the dilute acid pretreatment, the hydronium ions released by the acid resulted in depolymerization of hemicelluloses by selective hydrolysis of glycosidic linkages, liberating *O*-acetyl group and other acid moieties to form acetic and uronic acids. These acids released are thought to catalyze the hydrolysis of hemicelluloses and oligosaccharides. For control substrate (control-S, without dilute acid pretreatment), the characteristic bands of acetyl ester units of hemicelluloses at 1728 (C=O conjugates) and 1246 cm^−1^ (C–O) were observed by FT-IR spectra. As elevating severity, the intensities of the two bands were gradually reduced due to the deacetylation of hemicelluloses during the pretreatment [24]. When the pretreatment temperature was higher than 130 °C under the acid conditions, the two bands almost disappeared, confirming the significant degradation of hemicelluloses. During the dilute acid pretreatment, most of hemicelluloses were degraded, while lignin was retained in the pretreated substrates. In fact, most lignin embeds in the spaces of the plant cell walls and enhances the mechanical strength of the cell walls through covalently linked hemicelluloses. Previous literature reported that lignin had negative effects on the access of enzymes to cellulose [25]. In order to improve the glucose yield of biomass, an appropriate pretreatment should be employed to remove lignin from the pretreated substrates. Alkali solutions, such as aqueous NaOH, are widely used to disrupt the rigid structure of the cell wall for the separation of the main components of biomass. Wen et al. [26] demonstrated the yield, purity, dissociation mechanisms, and structural features of dissolved lignin (DL), milled wood lignin (MWL), and alkali lignin (AL) of the bamboo, and their results showed that the yield of AL was significantly higher than those of DL and MWL. In addition, Xiao et al. [27] also pointed out that solution of 1 % NaOH at 75 °C for 3 h was an effective method to disrupt the recalcitrant nature of the plant cell wall. Therefore, based on the above investigations, a further treatment was performed with 1.5 % aqueous NaOH at 80 °C for 3 h to isolate lignin from the pretreated substrates. As shown in Table 2, the contents of hemicelluloses and lignins unceasingly decreased in the post-treated substrates as compared to the pretreated substrates alone, further verifying that the delignification with alkali treatment effectively removed lignins and the residual hemicelluloses from the pretreated substrates. Especially, after the post-treatment process, the removal of lignins can enhance the glucose yield of substrate. Meanwhile, the lignins obtained as byproducts can also be recovered for further utilization. Furthermore, the disappearance of the two bands at 1728 and 1246 cm^−1^ in the post-treated substrates revealed that alkali treatment completely cleaved the ester bands of hemicelluloses, such as acetyl and uronic ester groups [28].

**Table 1:** The conditions of dilute acid pretreatment of rice straw and the solid yields obtained

Entry	Temperature (°C)	Time (h)	H_2_SO_4_ (%)	Solid yield (%)
1	100	2	0.5	76.54
2	110	2	0.5	72.01
3	120	2	0.5	68.06
4,10,14	130	2	0.5	64.65
5	140	2	0.5	63.58
6	150	2	0.5	61.62
7	130	0.5	0.5	65.16
8	130	1.0	0.5	65.12
9	130	1.5	0.5	64.69
11	130	2.5	0.5	62.68
12	130	3.0	0.5	60.10
13	130	2.0	0.25	66.91
15	130	2.0	0.6	62.28
16	130	2.0	0.7	61.73
17	130	2.0	0.8	59.91
18	130	2.0	1.0	56.15

**Table 2:** Chemical compositions of the control, pretreated, and post-treated substrates under various conditions

	Chemical composition (w/w, %)^a^				Chemical composition (w/w, %)^a^		
	Hemi^b^	Cellulose	Lignin		Hemi^b^	Cellulose	Lignin
Control-S	27.46	38.29	20.50	Control-R	33.57	56.45	3.00
S_(100-0.5-2)_	14.95	48.22	26.90	R_(100-0.5-2)_	13.03	78.37	2.53
S_(110-0.5-2)_	12.61	50.25	28.40	R_(110-0.5-2)_	7.13	80.29	2.81
S_(120-0.5-2)_	7.56	51.86	30.63	R_(120-0.5-2)_	4.17	81.93	6.38
S_(130-0.5-2)_	5.18	53.51	31.47	R_(130-0.5-2)_	3.14	82.29	7.23
S_(140-0.5-2)_	4.39	55.04	31.93	R_(140-0.5-2)_	2.26	82.81	7.89
S_(150-0.5-2)_	2.36	57.16	32.80	R_(150-0.5-2)_	1.03	84.29	8.31
S_(130-0.5-0.5)_	8.08	51.84	30.33	R_(130-0.5-0.5)_	5.32	79.34	5.30
S_(130-0.5-1)_	6.96	52.10	31.33	R_(130-0.5-1)_	3.83	80.15	5.56
S_(130-0.5-1.5)_	5.91	52.79	31.32	R_(130-0.5-1.5)_	2.53	81.82	5.88
S_(130-0.5-2.5)_	4.09	55.02	32.73	R_(130-0.5-2.5)_	2.33	82.29	6.75
S_(130-0.5-3)_	3.49	57.14	33.13	R_(130-0.5-3)_	1.84	83.81	7.61
S_(130-0.25-2)_	8.51	52.71	29.87	R_(130-0.25-2)_	4.47	80.17	5.42
S_(130-0.6-2)_	5.15	54.51	31.93	R_(130-0.6-2)_	1.95	83.01	7.00
S_(130-0.7-2)_	3.44	59.04	32.30	R_(130-0.7-2)_	1.80	84.55	8.58
S_(130-0.8-2)_	2.93	56.82	33.50	R_(130-0.8-2)_	1.47	82.23	9.60
S_(130-1-2)_	1.87	29.08	59.61	R_(130-1-2)_	1.17	78.13	11.43

^a^Cellulose expressed as glucan; Hemicelluloses expressed as xylan + arabinan + galactan; Lignin expressed as acid insoluble lignin (Klason lignin) and acid soluble lignin. Data represented are the averages of the results from duplicated experiments

^b^Hemi expressed as Hemicelluloses

^c^S refers to dilute acid pretreated materials under different conditions and R refers to the NaOH post-treated substrates from the dilute acid pretreated rice straw

The morphological changes of the pretreated and post-treated substrates were measured by SEM images (Additional file 1: Figure S2). The control-S exhibited a smooth and rigid surface structure. By contrast, the surfaces of the pretreated substrates were broken into separated fibers or fiber bundles and appeared cracks on different levels. The reason for this was mainly that the degradation of hemicelluloses during the pretreatment opened up macropores. During the further alkali treatment, the surface morphology of the substrates became looser with wider separation of the fibers than the pretreated substrates alone. This was possible that most of lignins and residual hemicelluloses were removed during the alkali post-treatment. The removal of both hemicelluloses and lignins resulted in the release of a larger specific surface area, which favored the following enzymatic hydrolysis.

Crystallinity is an essential factor that reflects the hydrolysis properties of substrates. The crystalline region hinders cellulases accessing to the cell walls of fibers, resulting in the reduction of cellulose hydrolysis [29]. The crystallinity indexes (CrIs) of the pretreated and post-treated substrates were determined by XRD patterns (Additional file 1: Figure S3) and the results are listed in Table 3. As compared to the CrI of control-S (34.0 %), the CrIs of the pretreated substrates increased from 40.8 to 43.0 % with the increase of the pretreatment temperature, H_2_SO_4_ concentration, or time. This increasing trend was attributed to the raise of cellulose concentration in the pretreated substrates due to the degradation and removal of amorphous hemicelluloses during the pretreatment process. However, the CrIs of the pretreated substrates began to decrease under a harsh condition (130 °C, 1.0 % H_2_SO_4_, 2.0 h), which was probably related to the partial degradation of crystalline cellulose. As expected, the further alkali treatment resulted in higher CrI values in the post-treated substrates, which was ascribed to the removal of lignins during the alkali post-treatment. Bi et al. [30] also reported that sodium hydroxide treatment enhanced the CrI of sugarcane bagasse since partial amorphous constituents (hemicelluloses and lignins) were removed during the treatment process.

**Table 3:** The crystallinity indexes of the control, pretreated, and the cellulose-rich fractions obtained by the integrated treatment

	CrI (%)		CrI (%)
Control-S	34.0	Control-R	42.1
S_(100-0.5-2)_	40.8	R_(100-0.5-2)_	42.8
S_(130-0.5-2)_	43.0	R_(130-0.5-2)_	48.9
S_(150-0.5-2)_	41.7	R_(150-0.5-2)_	49.2
S_(130-0.5-0.5)_	40.3	R_(130-0.5-0.5)_	47.6
S_(130-0.5-3)_	41.2	R_(130-0.5-3)_	46.7
S_(130-0.25-2)_	42.8	R_(130-0.25-2)_	44.8
S_(130-1-2)_	42.5	R_(130-1-2)_	46.6

### Effects of dilute acid pretreatments on xylooligosaccharides and degraded byproducts of polysaccharides

**Fig. 2:**
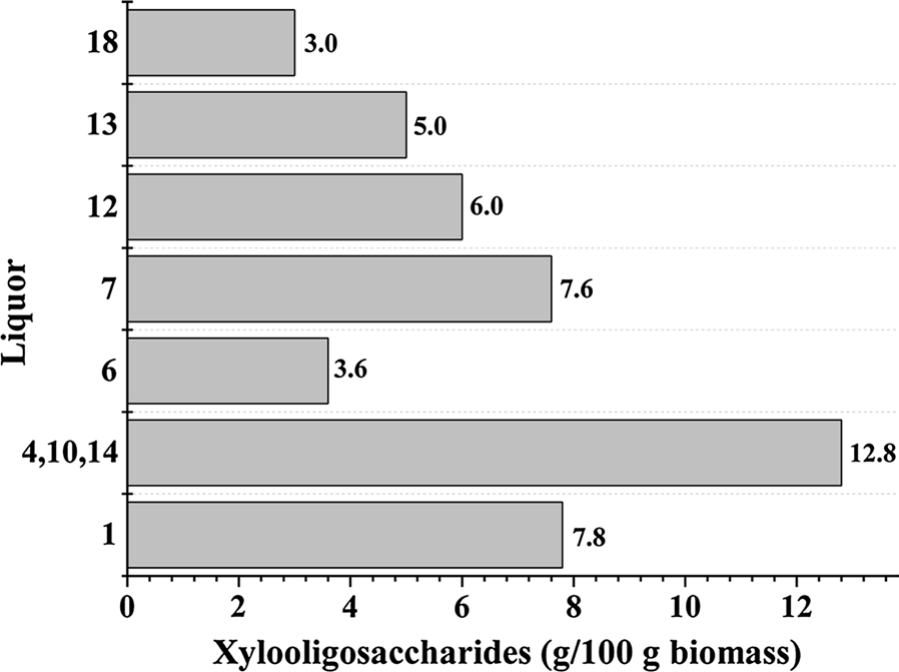
The yield of xylooligosaccharides in the liquor phase obtained during the various dilute acid pretreatments. The liquid number (1, 4, 10, 14, 6, 7, etc.) on the *y axis* correspond to the liquids obtained during the dilute acid pretreatment of rice straw under various conditions, and pretreatment conditions are given in detail in Table 1

The XOS production was influenced by the pretreatment conditions (Fig. 2). As the pretreatment temperature increased from 100 to 130 °C, the yields of XOS were raised from 7.8 to 12.8 g XOS/100 g RS. However, when the pretreatment temperature further increased to 150 °C, the yields of XOS rapidly declined (12.8–3.6 g XOS/100 g RS). These phenomena were probably that the XOS were further degraded into other small molecules (such as furfural) under the harsh conditions, as revealed by the subsequent degraded byproducts analysis in the liquors produced during the pretreatment. Likewise, when the reaction time was prolonged from 0.5 to 2.0 h at 130 °C with 0.5 % H_2_SO_4_, the yield of XOS increased. However, further prolonging the reaction time to 3.0 h, the yield of XOS rapidly reduced. Interestingly, the effect of dilute acid concentration on yield of XOS showed a similar tendency. It was found that a maximum yield of XOS was achieved (12.8 g XOS/100 g RS) at 130 °C with 0.5 % H_2_SO_4_ for 2.0 h in this study. Recently, a relatively high XOS production from oil palm empty fruit bunch by hot compressed water pretreatment was studied [31]. Results showed that the highest XOS yield of 4.8/100 g biomass was obtained at 190 °C for 20 min. As compared to hot compressed water treatment, the higher XOS yield was obtained by the dilute acid pretreatment under the optimized pretreatment condition in this study. Thus, dilute acid pretreatment is a promising method for the production of XOS from RS.

When XOS was produced by the dilute acid pretreatment, a variety of other compounds, such as formic acid, acetic acid, levulinic acid, lactic acid, hydroxymethylfurfural (HMF), and furfural was produced and appeared in the reaction media. The concentrations of the degraded byproducts of polysaccharides in the liquor phase are listed in Additional file 1: Table S1. It is interesting to note that the release of the degraded byproducts was closely related to the pretreatment conditions. For example, the concentrations of all the degraded byproducts increased with the growth of pretreatment temperature, H_2_SO_4_ concentration, or time. It was found that the maximum concentration of all the degraded byproducts was observed at 150 °C with 0.5 % H_2_SO_4_ for 2.0 h. This indicated that the pretreatment temperature was more effective than the H_2_SO_4_ concentration and reaction time on the degraded byproducts from the polysaccharides. Due to the existence of the degraded byproducts from polysaccharides during the dilute acid pretreatment, the liquor phase was not a very pure source of XOS. In industry, to purify and then produce commercial XOS, the liquors have to be refined by removing non-XOS compounds [32]. In the past two decades, several promising purification technologies have been developed, such as membrane technology, chromatographic separation, solvent extraction, and adsorption [33–36]. Among these technologies, membrane separation technology is the most promising technology for refining and concentrating XOS as compared to others, since they may be less expensive [33]. In this case, the size-dependent selection mechanism of the membrane process results in the various concentrations of molecules with different molecular weights. Membrane separations have been applied for the preparation of several XOS from various biomasses, such as Olive stones, *Pinus pinaster* wood, and forest waste [37–39].

### Effects of dilute acid pretreatments on the yield, purity, molecular weight, and the structure feature of the alkali lignins released

The yields of alkali lignins, which were calculated based on the lignins in respective substrate, are shown in Additional file 1: Table S2. The data showed that only 30.7 % of the lignin from the control-S was obtained. For the pretreated substrates, the yields of the lignins increased with the raise of pretreatment temperature, H_2_SO_4_ concentration, or time. The increasing trend was related to the dilute acid pretreatment conditions, which cleaved the chemical bonds between lignin and hemicelluloses to different degrees [40]. Thus, the pretreatment accelerated more lignins release (33.16–47.76 %) during the alkali post-treatment. In other words, the dilute acid pretreatment was a promising process for efficiently loosing the tight cell wall structure, and then facilitating the release of lignin from the plant cell wall with the assist of alkali treatment. Moreover, to verify the purity of the lignins isolated, the associated polysaccharides in the lignins were detected by HPAEC (Additional file 1: Table S2). The date showed that all the lignins contained rather low amounts of associated polysaccharides (<3.29 %). As compared to the control lignin, the amounts of polysaccharides in the pretreated lignins were reduced, suggesting that the hemicelluloses were significantly degraded during the pretreatment under the conditions given. To assess the effect of the pretreatment on the molecular weight of the lignins isolated, the lignins were performed by GPC. The changes in molecular weight of the lignins based on the different pretreatment conditions are listed in Additional file 1: Table S3. The molecular weight (*M*_w_) of control lignin was 2910 g mol^−1^, and its polydispersity (*M*_w_/*M*_n_) was 1.87. During the pretreatment, it was observed that the *M*_w_ (2320–2870 g mol^−1^) of the lignins reduced slightly. This suggested that the lignins underwent a slight depolymerization under the conditions given [41]. In addition, the polydispersity indexes (1.45–1.78) of the alkali lignins were reduced slightly, implying the formation of some homogeneous lignin fragments, which were probably originated from successive dilute acid and alkali treatments.

**Fig. 3:**
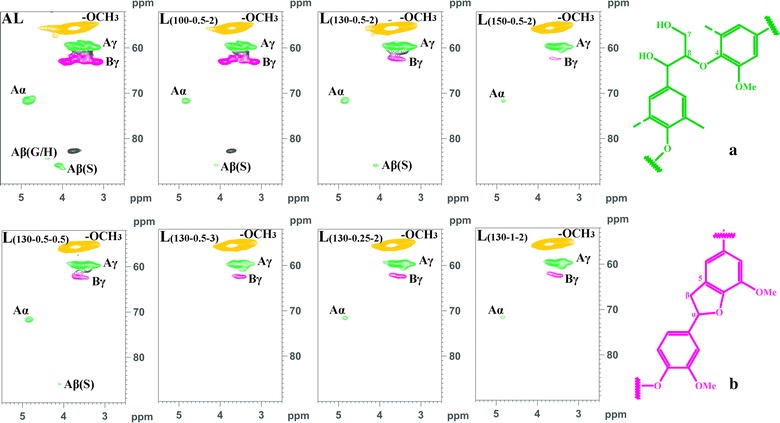
2D-HSQC spectra and the main structures of the lignins obtained from the integrated process under various processing conditions (side-chain region). *β*-aryl-ether units (**a** *β*-*O*-4) and phenylcoumaran substructures (**b** *β*-5)

**Fig. 4:**
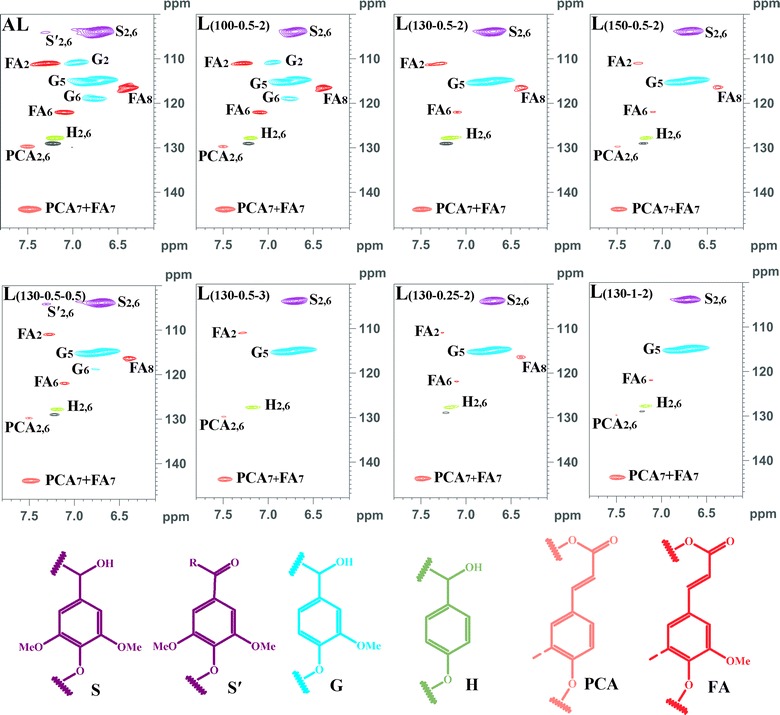
2D-HSQC spectra and the main structures of the lignin polymers obtained from the integrated process under the various processing conditions (aromatic region). *PCA* free *p*-coumaric acid; *FA* ferulic acid; *H p*-hydroxyphenyl units; *G* guaiacyl units; *S* syringyl units; *S*′ oxidized syringyl units bearing a carbonyl at C_*α*_

To further reveal the structural transformations of the lignins isolated during the various pretreatment conditions, the samples were investigated by 2D-HSQC NMR spectra (Figs. 3, 4) and the signals are assigned (Additional file 1: Table S4) [42–47]. The inter-unit linkages in the lignins, *β*-ether (A, *β*-*O*-4) and phenylcoumaran (B, *β*-5) are identified due to the presence of cross-peaks at *δ*_C_/*δ*_H_ 71.7/4.83 (A_*α*_), 59.7/3.54 (A_*γ*_), and 62.7/3.55 (B_*γ*_), respectively. The cross-peaks of methoxy groups in the lignins (−OCH_3_, *δ*_C_/*δ*_H_ 55.6/3.72) were clearly observed. The aromatic lignin units syringyl (S), guaiacyl (G), and *p*-hydroxyphenyl (H) units showed prominent correlations at *δ*_C_/*δ*_H_ 103.8/6.70 (S_2,6_), 110.7/6.95 (G_2_), 115.3/6.76 (G_5_), 118.9/6.76 (G_6_), and 127.9/7.20 (H_2,6_), respectively. Minor amounts of oxidized S units (S′) were detected due to the presence of a correlation at *δ*_C_/*δ*_H_ 106.0/7.30 (S′_2,6_). Moreover, the lignins obtained appeared to contain small amounts of *p*-hydroxycinnamates (*p*-coumaric and ferulic acids). The *p*-coumaric acid (PCA) was characterized by some relatively intense correlations at *δ*_C_/*δ*_H_ 129.7/7.50 (PCA_2,6_) and 143.9/7.48 (PCA_7_), while ferulic acid (FA) was found at *δ*_C_/*δ*_H_ 110.9/7.27 (FA_2_), 116.6.0/6.39 (FA_8_), and 143.9/7.48 (FA_7_). As can be seen, as the pretreatment temperature, H_2_SO_4_ concentration, or time increased, some changes appeared in the HSQC spectra of lignins. For example, the correlated signals of *β*-*O*-4 were diminished, and the correlated signals of PCA and FA were reduced simultaneously. Interestingly, the signal intensities of C_2_–H_2_ and C_6_–H_6_ in G unit were decreased gradually and disappeared under the harsh conditions, which is attributable to the fact that the condensation reaction probably occurred at C-2 and C-6 of G unit in lignin with the side chains of other lignin [48, 49].

In order to further investigate the effects of pretreatment conditions on the major hydroxyl groups of lignins, the lignins isolated were estimated by ^31^P NMR based on the integration area of individual peaks (Additional file 1: Figure S4). The contents of aliphatic hydroxyls, condensed and non-condensed syringyl and guaiacyl hydroxyls, *p*-hydroxybenzoate phenolic hydroxyls, and carboxylic acid hydroxyls were compared in different lignin fractions (Table 4) [50, 51]. The results showed that the increase of the pretreatment temperature, H_2_SO_4_ concentration, or time resulted in the decrease of the aliphatic OH groups. This indicated that the hydroxyl groups in the side chain of lignin were probably fragmented and eliminated under the harsh conditions [52]. On the contrary, the phenolic OH groups in syringyl, guaiacyl, *p*-hydroxyphenyl OH, and free *p*-coumaric acid units increased obviously. The increased phenolic OH groups were probably due to the cleavage of *β*-*O*-4 linkages under harsh conditions [53]. Similar observation has been reported in an autohydrolysis pretreatment of poplar [54]. It was noticed that harsh pretreatment conditions resulted in the slight increase of carboxyl groups, indicating that oxidation reaction occurred during the pretreatment. In this study, due to the cleavage of *β*-*O*-4 linkages during the synergetic treatment process, the lignin fractions with an increased phenolic OH groups had a potential application as biomaterials or biochemicals for industries such as lignin–phenol–formaldehyde resins and potential antioxidant [15, 55–59]. Ibrahim et al. [56] examined the influence of chemical properties of lignins as well as determine their suitability for partial incorporation into phenol formaldehyde resins. The results showed that higher content of phenolic hydroxyl groups in kraft lignin allowed the activation of free ring position which makes the kraft lignin more reactive toward formaldehyde than the soda lignin. In addition, lignins have phenolic hydroxyl group that can act as a potential antioxidant in the food industry, preventing the loss of food flavor, color, and active vitamin content [57–59]. Pan et al. [59] reported that the content of phenolic hydroxyl groups in lignin was positively correlated to antioxidant activities of lignin.

**Table 4:** Quantification of the functional groups (mmol/g) by quantitative ^31^P-NMR in the alkali lignins obtained by the integrated process

Lignin fractions	Aliphatic OH	Syringyl OH		Guaiacyl OH		H + P−OH	Carboxylic group	Total phenolic OH
		C	NC	C	NC			
AL	2.72	0.02	0.12	0.07	0.35	0.16	0.86	0.72
L_(100-0.5-2)_	1.56	0.11	0.34	0.20	0.59	0.31	0.93	1.55
L_(130-0.5-2)_	1.40	0.16	0.34	0.21	0.60	0.35	0.93	1.66
L_(150-0.5-2)_	1.39	0.17	0.35	0.22	0.60	0.37	1.01	1.71
L_(130-0.5-0.5)_	1.45	0.11	0.31	0.19	0.57	0.34	0.93	1.52
L_(130-0.5-3)_	1.39	0.19	0.36	0.22	0.62	0.37	0.97	1.76
L_(130-0.25-2)_	1.47	0.18	0.32	0.19	0.60	0.32	0.88	1.61
L_(130-1-2)_	1.37	0.20	0.37	0.26	0.68	0.42	1.06	1.93

*C* condensed; *NC* non-condensed; *H* + *P*−*OH* the total content of *p*-hydroxyphenyl OH and free *p*-coumaric acid

### Effects of successive dilute acid and alkali treatments on the enzymatic hydrolysis of cellulose in the substrates

**Fig. 5:**
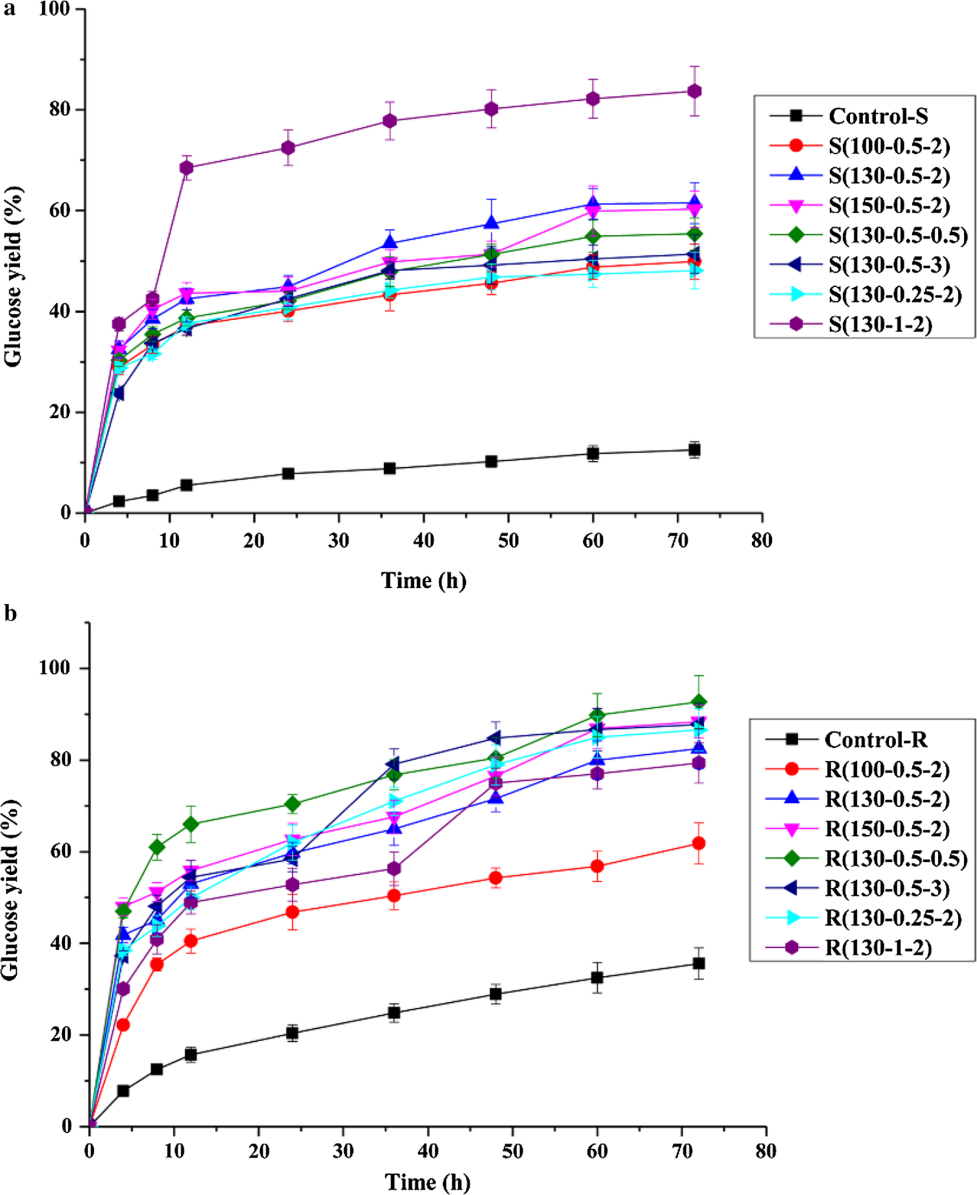
Glucose yields of enzymatic hydrolysis of the control, pretreated (**a**), and post-treated substrates (**b**). *Control-S* extractive-free raw material, *S* substrates obtained by the direct dilute acid pretreated materials under different conditions, *Control-R* substrate obtained by the alkali treatment raw material without dilute acid pretreatment, *R* substrates obtained by the alkali post-treated substrates from the dilute acid pretreated substrates

The performance of the enzymatic hydrolysis was assessed by determining glucose yield from the conversion of cellulose and this was expressed as the percentage of glucose released in relation to the total content of potential glucose in the starting material (i.e., the control, pretreated, and the post-treated substrates). Figure 5 shows the glucose yield of the control, pretreated (Fig. 5a), and post-treated substrates (Fig. 5b). As shown in Fig. 5a, the pretreatment on the glucose yield of the substrates increased by 37.4–71.2 % as compared with control substrate (12.5 %). A possible reason was that the pretreatment resulted in increased enzyme accessibility due to significant removal of hemicelluloses as well as damage of surface morphology of the substrates. The glucose yield of the alkali-treated substrate without dilute acid pretreatment was only 35.6 %, while the glucose yield of the pretreated and post-treated substrates increased to 61.8–92.7 %. Thus, the combining of dilute acid and alkali treatments was highly effective for removing hemicelluloses and lignin from RS, resulting in increased enzymatic accessibility of the substrate and more efficient enzymatic hydrolysis. However, in the present study, there were no direct correlations between glucose yields and the residual contents of lignin or hemicelluloses in the pretreated and post-treated substrates. Meanwhile, it was found that crystallinity was not an important factor for the enzymatic hydrolysis of the substrates. The results indicated that there was no clear relationship between the CrI and glucose yield since the cellulose hydrolysis was not only affected by cellulose crystallinity but also by several other factors, such as contents and distribution of lignins or hemicelluloses, particle size, and porosity of the substrates. Thus, single factor may not adequately elaborate the differences of hydrolysis, and several factors impacted the cellulose hydrolysis of the substrates in the current study.

### Effects of successive dilute acid and alkali treatments on the higher value application of rice straw

**Fig. 6:**
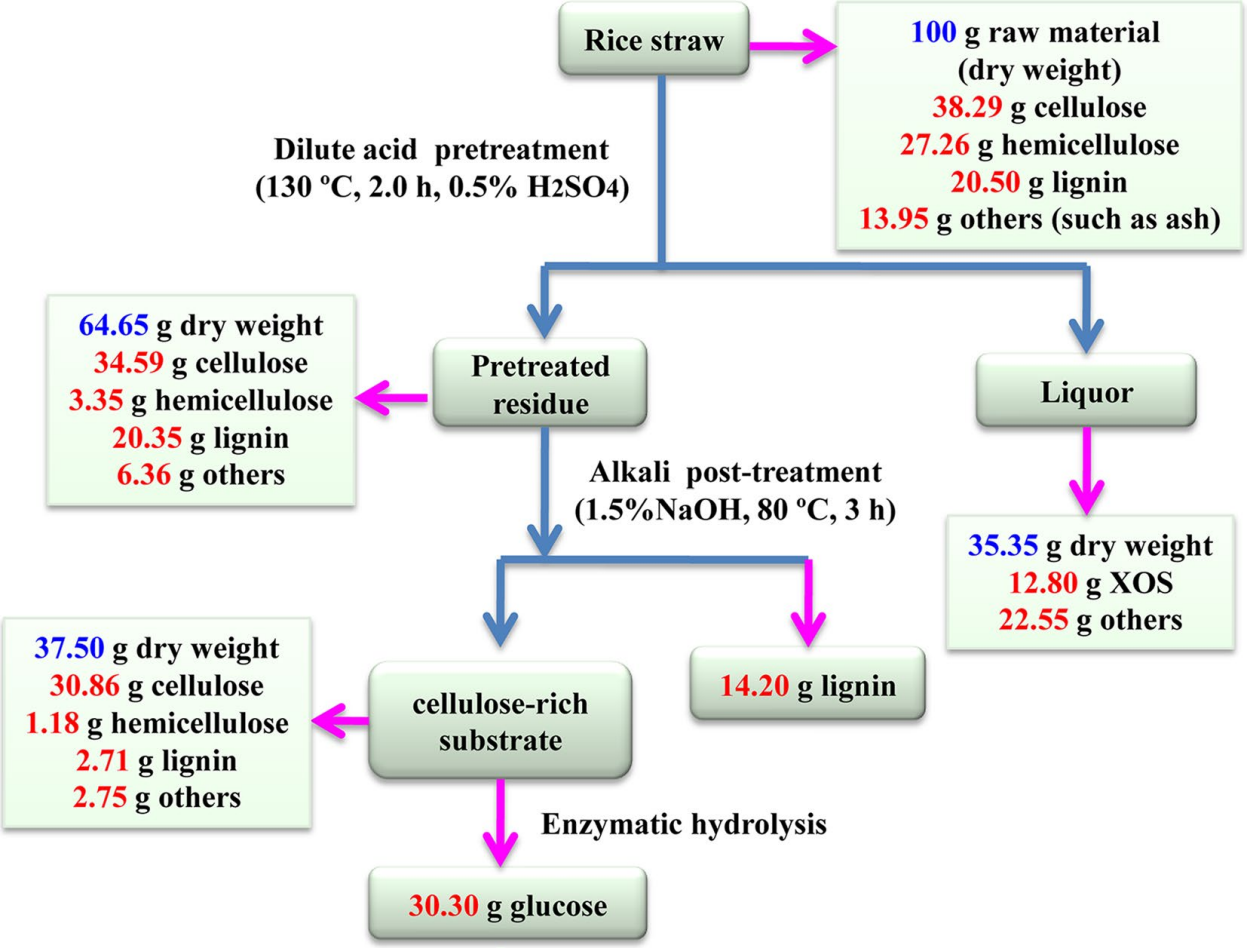
Mass balance during the pretreatment at 130 °C with 0.5 % sulfuric acid for 2.0 h

The maximum yield of XOS (12.8 g XOS/100 g RS) was obtained with a relatively low level of byproducts under the conditions of 130 °C with 0.5 % H_2_SO_4_ for 2.0 h. Enhancement of the pretreatment time, temperature, or H_2_SO_4_ concentration reduced the yield of XOS and increased the concentrations of the byproducts. Based on the above process condition, during the further alkali post-treatment, a relatively high yield of lignin (45.58 %) and cellulose-rich substrate (82.29 % cellulose) were collected. For the cellulose-rich substrate, the glucose yield reached to 88.4 % after enzymatic hydrolysis for 72 h. Process yield was normalized to a common basis of 100 g of dried RS as the starting material (Fig. 6). By the biorefinery process, hemicelluloses, lignins, and cellulose of RS were effectively separated and utilized. Specifically, 12.8 g XOS was obtained, meanwhile, 14.2 g lignin and 30.3 g glucose were also collected. Zhu et al. [60] reported that the integration of autohydrolysis and organosolv pretreatment led to an enhanced digestibility of *Eucommia ulmoides* Oliver wood as compared to autohydrolysis or organosolv pretreatment alone. Under the condition of autohydrolysis at 180 °C for 0.5 h and sequent treatment using 50 % aqueous ethanol with 1 % HCl as a catalyst, 9.5 g XOS, 14.5 g lignin, and 27.1 g glucose based on 100 g of initial biomass were obtained. Therefore, the biorefinery process based on successive dilute acid and alkali treatments for higher value application of rice straw is promising in industry.

## Conclusions

Successive treatments with dilute acid and alkali have been performed for the production of XOS, lignin, and cellulose-rich substrates for bioethanol production from rice straw. During the dilute acid pretreatment, a maximum production of XOS (12.8 g XOS/100 g RS) with a relatively low level of byproducts was obtained at 130 °C with 0.5 % H_2_SO_4_ for 2.0 h. On further alkali treatment, 14.2 g lignin with a higher purity of 99.2 % and 30.3 g glucose by enzymatic hydrolysis were also obtained from the successively treated substrates per 100 g starting material. The cleavage of *β*-*O*-4 linkages of lignin resulted in an increase of the content of phenolic OH groups with the elevating severity. During the treatment processes, the C-2 and C-6 of guaiacyl were condensed with the side chains of other lignin. The successive treatments of rice straw for production of XOS, lignin, and glucose can be further extended to the value-added application of various lignocellulosic biomasses.

## Methods

### Raw materials

RS was obtained from Shandong Province of China. RS was firstly dried in an oven at 60 °C for 24 h and then ground in a mill to obtain a 40–60 mesh powder. Then, the powder was extracted with toluene–ethanol (2:1, v/v) in a Soxhlet apparatus for 6 h to remove extractives and then dried in an oven at 60 °C for 24 h to serve as control substrate (control-S). The chemical composition of control-S (%, w/w) was determined to be cellulose 38.29 %, hemicelluloses 27.46 %, and lignin 20.50 %, according to National Renewable Energy Laboratory’s (NREL) standard analytical procedure. All chemicals were analytical grade and purchased from Sigma–Aldrich and Megazyme.

### Biorefinery process

Control-S was successively treated by dilute acid and alkali. The dilute acid pretreatments (100, 110, 120, 130, 140, and 150 °C with 0.5 % H_2_SO_4_ for 1.0 h; 130 °C with 0.5 % H_2_SO_4_ for 0.5, 1.0, 1.5, 2.5, and 3.0 h; and 130 °C with 0.25, 0.6, 0.7, 0.8, and 1.0 % H_2_SO_4_ for 2.0 h, respectively) were performed in a batch reactor (100 mL internal volume, Sen Long Instruments Company, Beijing, China) at a solid-to-liquor ratio of 1:15 (g/mL). The reactor was made of Hastelloy C-276 to mitigate the effects of acid corrosion at high temperatures. The substrates pretreated with dilute acid were filtered off with a Buchner funnel, washed thoroughly with distilled water, and further dried in a cabinet oven with air circulation at 60 °C for 16 h and then labeled as S_(100-0.5-2)_, S_(110-0.5-2)_, S_(120-0.5-2)_, S_(130-0.5-2)_, S_(140-0.5-2)_, S_(150-0.5-2)_, S_(130-0.5-0.5)_, S_(130-0.5-1)_, S_(130-0.5-1.5)_, S_(130-0.5-2.5)_, S_(130-0.5-3)_, S_(130-0.25-2)_, S_(130-0.6-2)_, S_(130-0.7-2)_, S_(130-0.8-2)_, and S_(130-1-2)_, respectively, corresponding to temperature, H_2_SO_4_ concentration, and time of pretreatment.

A further treatment was performed with 1.5 % aqueous NaOH at 80 °C for 3 h under a solid-to-liquid ratio of 1:10 (g/mL) to isolate lignin from the pretreated substrates and obtain post-treated substrates (cellulose-rich substrates). The collected liquid fractions were concentrated to 40 mL and poured into 200 mL of acidic water (pH 2.0, adjusted by HCl) to precipitate the alkali-soluble lignins. The precipitations were centrifuged, freeze-dried, and then named as L_(100-0.5-2)_, L_(110-0.5-2)_, L_(120-0.5-2)_, L_(130-0.5-2)_, L_(140-0.5-2)_, L_(150-0.5-2)_, L_(130-0.5-0.5)_, L_(130-0.5-1)_, L_(130-0.5-1.5)_, L_(130-0.5-2.5)_, L_(130-0.5-3)_, L_(130-0.25-2)_, L_(130-0.6-2)_, L_(130-0.7-2)_, L_(130-0.8-2)_, and L_(130-1-2)_ according to the pretreatment conditions of the temperature, the H_2_SO_4_ concentration, and the time. Control lignin (i.e., alkaline lignin, AL) was also isolated from the control-S under the same alkali treatment condition. Meanwhile, the alkaline post-treated substrates obtained from the corresponding pretreated substrates were filtered off with a Buchner funnel, washed thoroughly with distilled water, and further dried in a cabinet oven with air circulation at 60 °C for 16 h and then labeled as R_(100-0.5-2)_, R_(110-0.5-2)_, R_(120-0.5-2)_, R_(130-0.5-2)_, R_(140-0.5-2)_, R_(150-0.5-2)_, R_(130-0.5-0.5)_, R_(130-0.5-1)_, R_(130-0.5-1.5)_, R_(130-0.5-2.5)_, R_(130-0.5-3)_, R_(130-0.25-2)_, R_(130-0.6-2)_, R_(130-0.7-2)_, R_(130-0.8-2)_, and R_(130-1-2)_, respectively. As a control substrate, control-R was also obtained from control-S under the same alkali treatment condition without dilute acid pretreatment. All of the prepared substrates (control, pretreated, and combining alkaline post-treated) were used to produce glucose by enzymatic hydrolysis in the present study.

### Enzymatic hrdrolysis

Enzymatic hydrolysis was performed on the substrate (2 % w/v) in 10 mL 50 mM sodium acetate buffer (pH 4.8) with a 25 mL Erlenmeyer flask at 50 °C in a double-layer shaking incubators (ZWYR-2102C) (Shanghai, China) at 150 rpm for 72 h. Commercial enzyme (Cellic^®^CTec2, 17 FPU/g substrate) was provided by Novozymes North America, Inc. (Franklinton, NC) and employed for all the enzymatic hydrolyses. The hydrolyzates were sampled periodically and analyzed by a HPAEC (Dionex, ICS 3000, U.S.) system equipped with an integral amperometric detector and CarboPac PA 100 (4 × 250 mm, Dionex) analytical column. The detailed procedures were previously described in a publication [61]. All of the hydrolysis experiments were performed in triplicate, and average values and corresponding derivations were given.

### Analysis methods

The chemical compositions (%, w/w) of all the substrates were determined by NREL standard analytical procedure. The content of cellulose and hemicelluloses in the substrates was determined by HPAEC system on a CarboPac PA20 (3 × 150 mm, Dionex) analytical column. SEM images were performed with a S-3400 N II (Hitachi, Japan) instrument at 10 kV and 81 mA. FT-IR spectroscopic measurements were conducted on a Nicolet iN10 spectrophotometer in the range of 1760–860 cm^−1^ at 4 cm^−1^ resolution with 64 scans. XRD in reflection mode was performed using an XRD-6000 apparatus (Shimadzu, Japan) with Ni-filtered Cu K_*α*_ radiation (*χ* = 1.54 Å) generated at 40 kV and 30 mA. The scattering angle (2*θ*) was from 10 to 36° at a scanning speed of 2°/min. The CrIs of the substrates were calculated from the ratio of the crystalline peak area to the total area of crystalline and amorphous peaks.

The liquor obtained from dilute acid pretreatment was stored and filtered to determine XOS and byproducts (e.g., formic acid, furfural). Liquor sample of 3 mL was post-hydrolyzed with 4 % H_2_SO_4_ at 121 °C for 1 h to determine the total concentration of XOS. The increased concentrations of monosaccharides after post-hydrolysis were related to the concentration of XOS [62]. According to this procedure, saccharides of DP 2 or higher were considered as XOS. All the liquor fractions were filtered through 0.22-μm filter and subsequently analyzed by HPAEC system. The amounts of byproducts in the liquid fractions were also quantitatively determined by high-performance liquid chromatography (HPLC; Agilent 1200, USA) with a Bio-Rad Aminex HPX-87H analytical column and a refractive index detector. The eluent was 5 mM H_2_SO_4_ solution with a volumetric flow rate of 0.5 mL/min. Column temperature was set at 50 °C. The temperature of RI detector was 40 °C and injection volume was 10 μL.

The associated polysaccharides in the lignins were calculated using HPAEC as reported previously [13]. Molecular weights of the lignins were determined by GPC with an ultraviolet detector at 240 nm. The column used was a PL-gel 10 mm mixed-B 7.5 mm i.d. column, which was calibrated with PL polystyrene standards. Four milligrams of the lignins were dissolved in 2 mL of tetrahydrofuran (THF), and 20 μL of lignin solutions was injected. The column was operated at ambient temperature and eluted with THF at a flow rate of 1.0 mL/min. The solution-state 2D HSQC spectra of the lignins were acquired on a Bruker AVIII 400 MHz spectrometer at 25 °C. The data were acquired using 60 mg of lignin in 0.5 mL of DMSO-*d*_6_. Functional groups (phenolic hydroxyl, aliphatic hydroxyl, and carboxyl groups) of the lignins were determined by ^31^P NMR spectra according to a previous publication [63].

## Additional file

**Additional file 1: Table S1.** The byproducts liberated during the dilute acid pretreatment. **Table S2.** Yield and contents of associated polysaccharides (absolute %, w/w) of the lignins isolated with 1.5 % NaOH at 80 °C for 2 h from the dilute acid pretreated rice straw. **Table S3.** Weight-average (*Μ*_*w*_) and number-average (*Μ*_*n*_) molecular weights, and polydispersity (*Μ*_*w*_ */Μ*_*n*_) of the alkaline lignin fractions. **Table S4.** Assignments of ^13^C-^1^H cross-peaks in HSQC spectra of the alkaline lignins recovered from the integrated process. **Figure S1.** FT-IR spectra of the raw material, acid pretreated straw and alkali post-treated straw. **Figure S2.** SEM images of the raw material, acid pretreated straw and alkali post-treated straw. **Figure S3.** XRD patterns of the raw material, acid pretreated straw and alkali post-treated straw. **Figure S4.** ^31^P-NMR spectra of the lignins obtained from the integrated process under various processing conditions.

## Abbreviations

XOS: xylooligosaccharides
RS: rice straw
HPAEC: high-performance anion-exchange chromatography
SEM: scanning electron microscopy
FT-IR: fourier transform infrared
XRD: X-ray diffraction
NREL: National Renewable Energy Laboratory’s
CrI: crystallinity index
HPLC: high-performance liquid chromatography
DP: degree of polymerization
GPC: gel permeation chromatography
THF: tetrahydrofuran
2D-HSQC: two-dimensional heteronuclear single quantum coherence
HMF: hydroxymethylfurfural
*M*_*w*_: weight-average
*M*_*n*_: number-average
WSP: water-soluble polysaccharide
S: syringyl
S′: oxidized syringyl
G: guaiacyl
H: *p*-hydroxyphenyl
PCA: free *p*-coumaric acid
FA: ferulic acid
