# Supplementary material for: Synergetic effect of dilute acid and alkali treatments on fractional application of rice straw
Source: Biotechnol Biofuels. 2016 Oct 18;9:217. doi: 10.1186/s13068-016-0632-9 (PMC5069894; doi:10.1186/s13068-016-0632-9)

**Synergetic effect of dilute acid and alkali treatments on fractional application of rice** **straw**

Shaolong Sun

Email: sunshaolong328@126.com

Weijing Chen

Email: bjfu130544228@163.com

Jianing Tang

Email: 524917373@qq.com

Bing Wang

Email: wangbing4758@163.com

Xuefei Cao

Email: caoxuefei@bjfu.edu.cn

Shaoni Sun^*^

Email: sunshaoni@126.com

Run-Cang Sun^*^

Email: rcsun3@bjfu.edu.cn

Beijing Key Laboratory of Lignocellulosic Chemistry, Beijing Forestry University, Beijing, 100083, China

*Corresponding authors, *Phone: +86-10-62336972.

Fax: +86-10-62336972. E-mail: rcsun3@bjfu.edu.cn.

*Phone: +86-10-62336592.

Fax: +86-10-62336592. E-mail: sunshaoni@126.com.

**Table S1**

**The byproducts liberated during the dilute acid pretreatment**

| **Samples** | **Byproducts liberated during dilute acid pretreatment (g/L)** | | | | | |
| --- | --- | --- | --- | --- | --- | --- |
|  | **Formic acid** | **Acetic acid** | **Levulinic acid** | **Lactic acid** | **HMF** | **Furfural** |
| 1 | 0.05 | 0.04 | 0.00 | 1.23 | 0.00 | 0.00 |
| 2 | 0.11 | 0.04 | 0.02 | 1.47 | 0.00 | 0.00 |
| 3 | 0.25 | 0.04 | 0.02 | 1.72 | 0.00 | 0.00 |
| 4 ,10,14 | 0.26 | 0.08 | 0.03 | 1.86 | 0.02 | 1.11 |
| 5 | 0.43 | 0.15 | 0.07 | 1.96 | 0.12 | 2.78 |
| 6 | 0.65 | 0.20 | 0.13 | 2.11 | 0.31 | 2.89 |
| 7 | 0.01 | 0.04 | 0.00 | 1.72 | 0.00 | 0.02 |
| 8 | 0.08 | 0.07 | 0.02 | 1.80 | 0.00 | 0.56 |
| 9 | 0.24 | 0.08 | 0.02 | 1.84 | 0.00 | 0.97 |
| 11 | 0.38 | 0.08 | 0.04 | 1.91 | 0.03 | 1.32 |
| 12 | 0.44 | 0.16 | 0.05 | 2.06 | 0.07 | 2.23 |
| 13 | 0.26 | 0.08 | 0.02 | 1.68 | 0.00 | 0.00 |
| 15 | 0.28 | 0.12 | 0.03 | 1.71 | 0.02 | 1.25 |
| 16 | 0.28 | 0.12 | 0.06 | 1.87 | 0.04 | 1.44 |
| 17 | 0.31 | 0.13 | 0.08 | 1.93 | 0.07 | 1.68 |
| 18 | 0.34 | 0.16 | 0.10 | 2.01 | 0.07 | 1.89 |

ND, not detected.

**Table S2**

**Yield and contents of associated polysaccharides (absolute %, w/w) of the lignins isolated with 1.5% NaOH at 80 ºC for 2 h from the dilute acid** pretreated rice straw

|  | **Yield^a^** | **Arabinose** | **Galactose** | **Glucose** | **Xylose** | **Total sugars** |
| --- | --- | --- | --- | --- | --- | --- |
| AL | 27.51 | 0.80 | 0.25 | 0.21 | 2.03 | 3.29 |
| L_(100-0.5-2)_ | 33.16 | 0.17 | 0.13 | 0.55 | 0.33 | 1.18 |
| L_(110-0.5-2)_ | 39.75 | 0.18 | 0.06 | 0.52 | 0.72 | 1.49 |
| L_(120-0.5-2)_ | 44.18 | 0.18 | 0.00 | 0.62 | 0.19 | 0.99 |
| L_(130-0.5-2)_ | 45.28 | 0.12 | 0.00 | 0.58 | 0.13 | 0.83 |
| L_(140-0.5-2)_ | 46.33 | 0.09 | 0.00 | 0.58 | 0.09 | 0.77 |
| L_(150-0.5-2)_ | 47.56 | 0.13 | 0.00 | 0.56 | 0.22 | 0.91 |
| L_(130-0.5-0.5)_ | 38.36 | 0.19 | 0.00 | 0.47 | 0.18 | 0.85 |
| L_(130-0.5-1)_ | 38.78 | 0.18 | 0.00 | 0.48 | 0.15 | 0.82 |
| L_(130-0.5-1.5)_ | 41.10 | 0.18 | 0.00 | 0.81 | 0.18 | 1.17 |
| L_(130-0.5-2.5)_ | 46.10 | 0.14 | 0.00 | 0.69 | 0.26 | 1.09 |
| L_(130-0.5-3)_ | 47.22 | 0.12 | 0.00 | 1.32 | 0.13 | 1.57 |
| L_(130-0.25-2)_ | 43.90 | 0.14 | 0.00 | 1.83 | 0.22 | 2.19 |
| L_(130-0.6-2)_ | 45.46 | 0.18 | 0.00 | 0.54 | 0.12 | 0.83 |
| L_(130-0.7-2)_ | 45.96 | 0.17 | 0.00 | 0.77 | 0.14 | 1.08 |
| L_(130-0.8-2)_ | 46.54 | 0.18 | 0.00 | 1.43 | 0.17 | 1.77 |
| L_(130-1-2)_ | 47.76 | 0.21 | 0.00 | 0.72 | 0.10 | 1.03 |

^a^ Based on the lignin content in the corresponding rice straw (%, w/w).

**Table S3**

**Weight-average (*Μ_w_*) and number-average (*Μ_n_*) molecular weights, and polydispersity (*Μ_w_/Μ_n_*)**

**of the alkaline lignin fractions**

|  | ***Μ_w_*** | ***Μ_n_*** | ***Μ_w_/Μ_n_*** |
| --- | --- | --- | --- |
| AL | 2910 | 1550 | 1.87 |
| L_(100-0.5-2)_ | 2320 | 1310 | 1.78 |
| L_(110-0.5-2)_ | 2560 | 1470 | 1.74 |
| L_(120-0.5-2)_ | 2770 | 1530 | 1.81 |
| L_(130-0.5-2)_ | 2870 | 1810 | 1.59 |
| L_(140-0.5-2)_ | 2640 | 1660 | 1.58 |
| L_(150-0.5-2)_ | 2540 | 1610 | 1.58 |
| L_(130-0.5-0.5)_ | 2400 | 1480 | 1.63 |
| L_(130-0.5-1)_ | 2630 | 1680 | 1.57 |
| L_(130-0.5-1.5)_ | 2690 | 1710 | 1.57 |
| L_(130-0.5-2.5)_ | 2830 | 1950 | 1.45 |
| L_(130-0.5-3)_ | 2770 | 1660 | 1.67 |
| L_(130-0.25-2)_ | 2580 | 1710 | 1.50 |
| L_(130-0.6-2)_ | 2870 | 1790 | 1.60 |
| L_(130-0.7-2)_ | 2830 | 1880 | 1.51 |
| L_(130-0.8-2)_ | 2660 | 1740 | 1.53 |
| L_(130-1-2)_ | 2640 | 1730 | 1.53 |

**Table S4**

**Assignments of ^13^C-^1^H cross-peaks in HSQC spectra of the alkaline lignins from the integrated process**

| Lable | *δ*_C_/*δ*_H_ (ppm) | Assignments |
| --- | --- | --- |
| –OCH_3_ | 55.6/3.72 | C−H in methoxyls |
| A*_γ_* | 59.7/3.54 | C*_γ_*−H*_γ_* in *β*-*O*-4′ substructures (A) |
| B*_γ_* | 62.7/3.55 | C*_γ_*−H*_γ_* in phenylcoumaran substructures (B) |
| A*_α_* | 71.7/4.83 | C*_α_*−H*_α_* in *β*-*O*-4′ units (A) |
| A*_β_*_(G/H)_ | 84.4/4.36 | C*_β_*−H*_β_* in *β*-*O*-4′ linked to G/H unit (A) |
| A*_β_*_(S)_ | 85.9/4.09 | C*_β_*−H*_β_* in *β*-*O*-4′ linked to a S unit (A) |
| S_2,6_ | 103.8/6.70 | C_2,6_−H_2,6_ in syringyl units (S) |
| S′_2,6_ | 104.1/7.30 | C_2,6_−H_2,6_ in oxidized (C*_α_*=O) S units (S′) |
| G_2_ | 110.7/6.95 | C_2_−H_2_ in guaiacyl units (G) |
| G_5_ | 115.3/6.76 | C_5_−H_5_ in guaiacyl units (G) |
| G_6_ | 118.9/6.76 | C_6_−H_6_ in guaiacyl units (G) |
| H_2,6_ | 127.9/7.20 | C_2,6_−H_2,6_ in H units (H) |
| PCA _2,6_ | 129.7/7.50 | C_2,6_−H_2,6_ in *p*-coumaric acid (PCA) |
| PCA_7_ | 143.9/7.48 | C_7_−H_7_ in *p*-coumaric acid (PCA) |
| FA_2_ | 110.9/7.27 | C_2_−H_2_ in ferulate (FA) |
| FA_6_ | 122.1/7.10 | C_6_−H_6_ in ferulate (FA) |
| FA_7_ | 143.9/7.48 | C_7_−H_7_ in ferulate (FA) |
| FA_8_ | 116.6/6.39 | C_8_−H_8_ in ferulate (FA) |

**Figure S1**

**FT-IR spectra of the raw material, acid pretreated straw and alkali post-treated straw.**


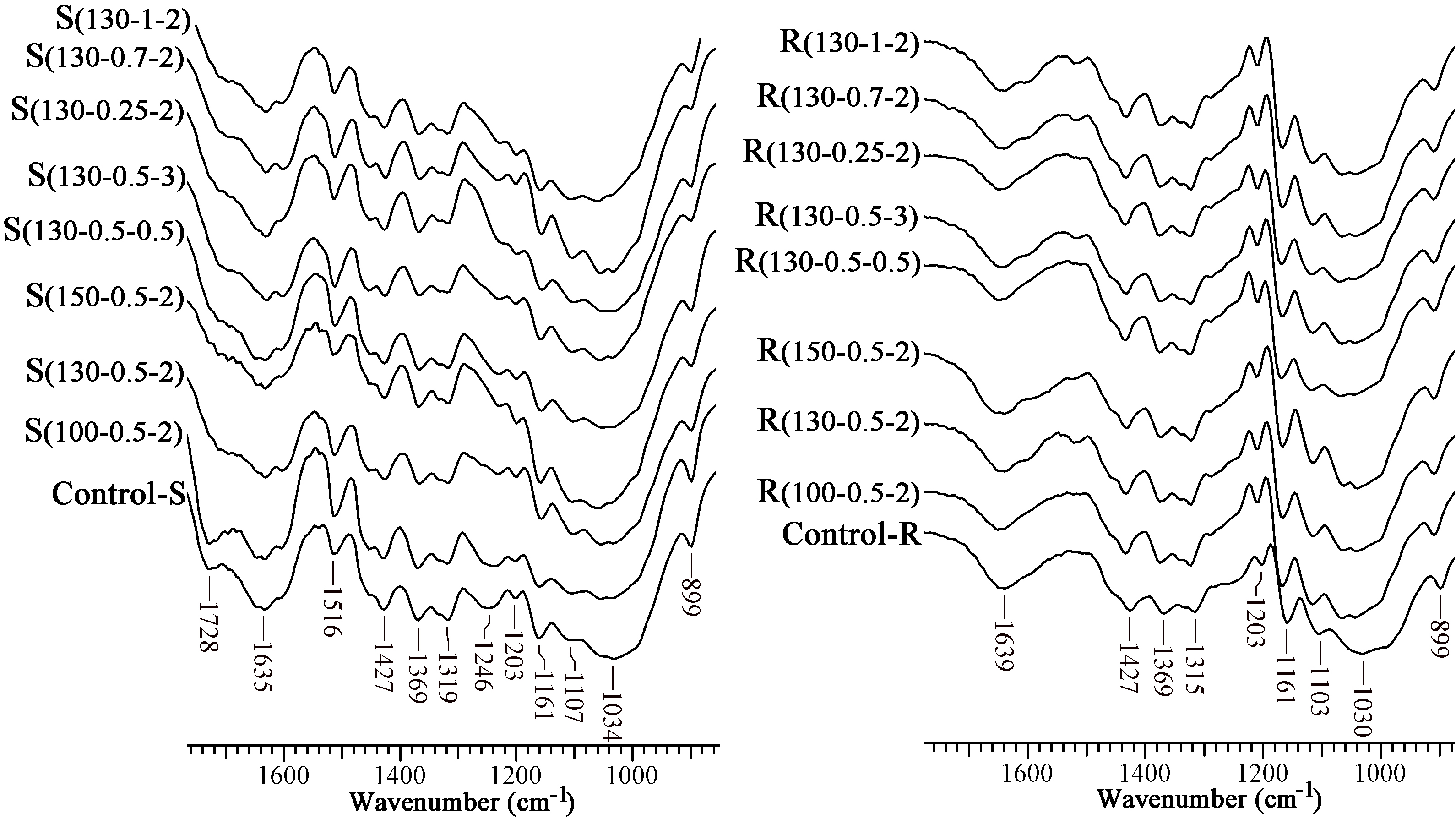


**Figure S2**

**SEM images of the raw material, acid pretreated straw and alkali post-treated straw.**


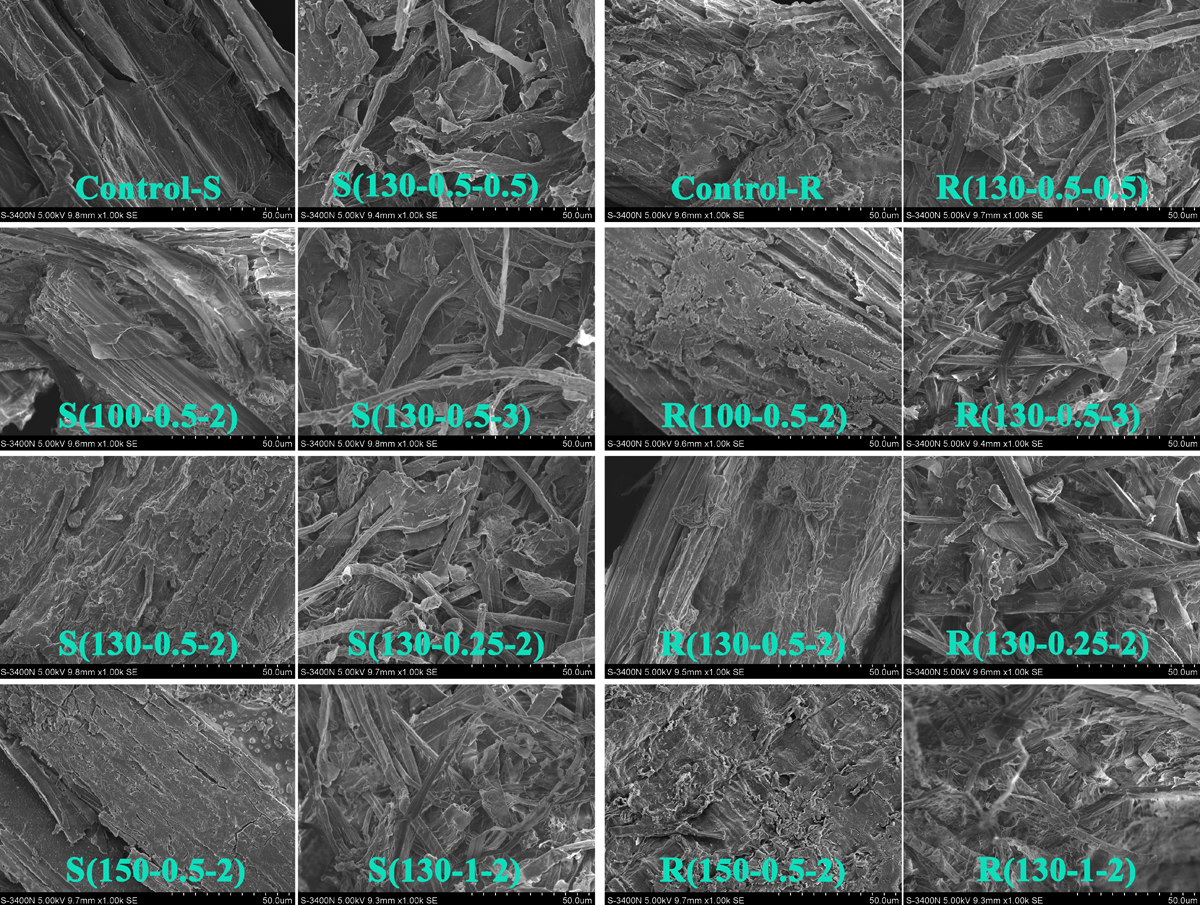


**Figure S3**

**XRD patterns of the raw material, acid pretreated straw and alkali post-treated straw.**


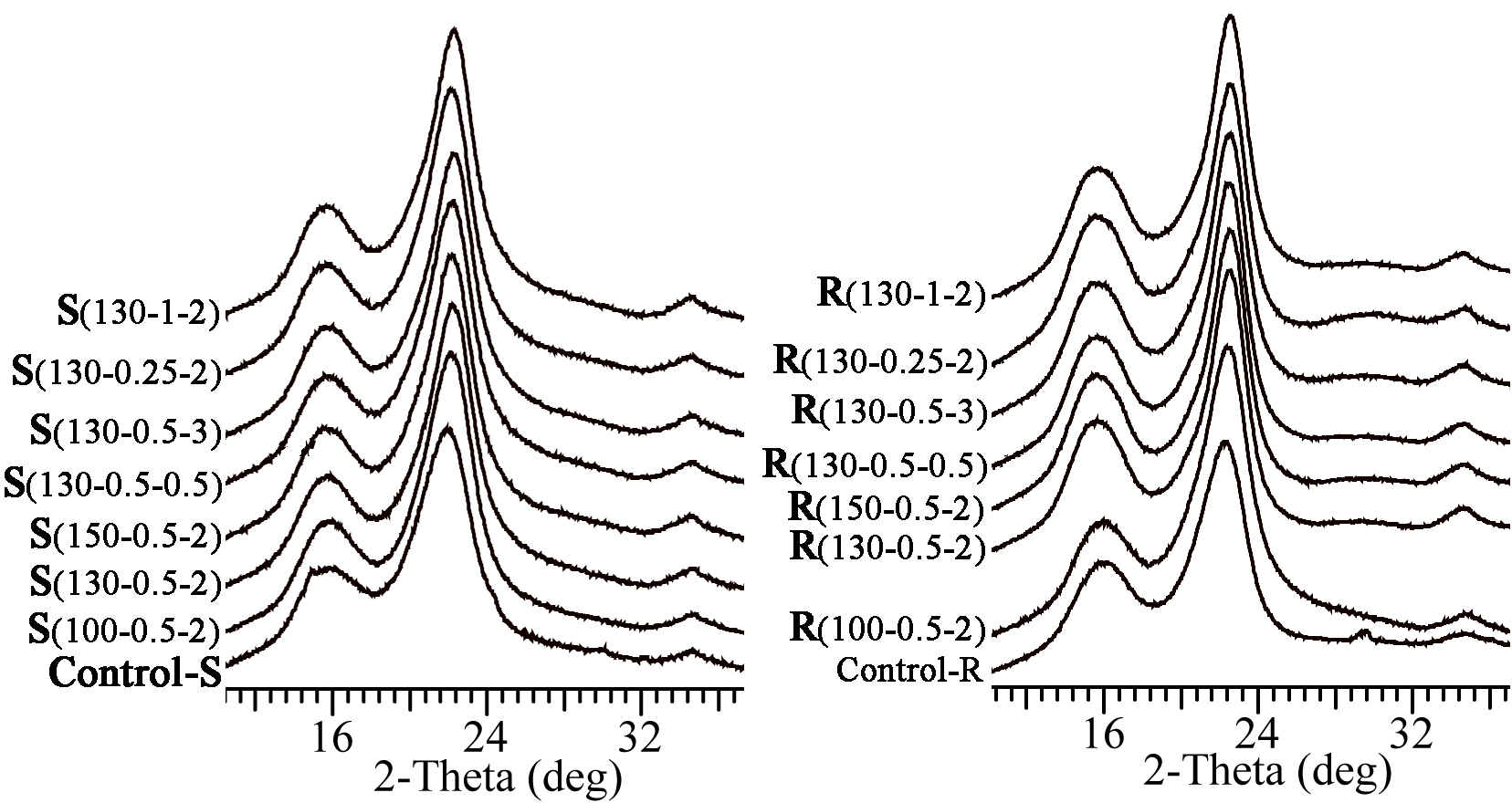


**Figure S4**

**^31^P-NMR spectra of the lignins obtained from the integrated process under various**

**processing conditions.**


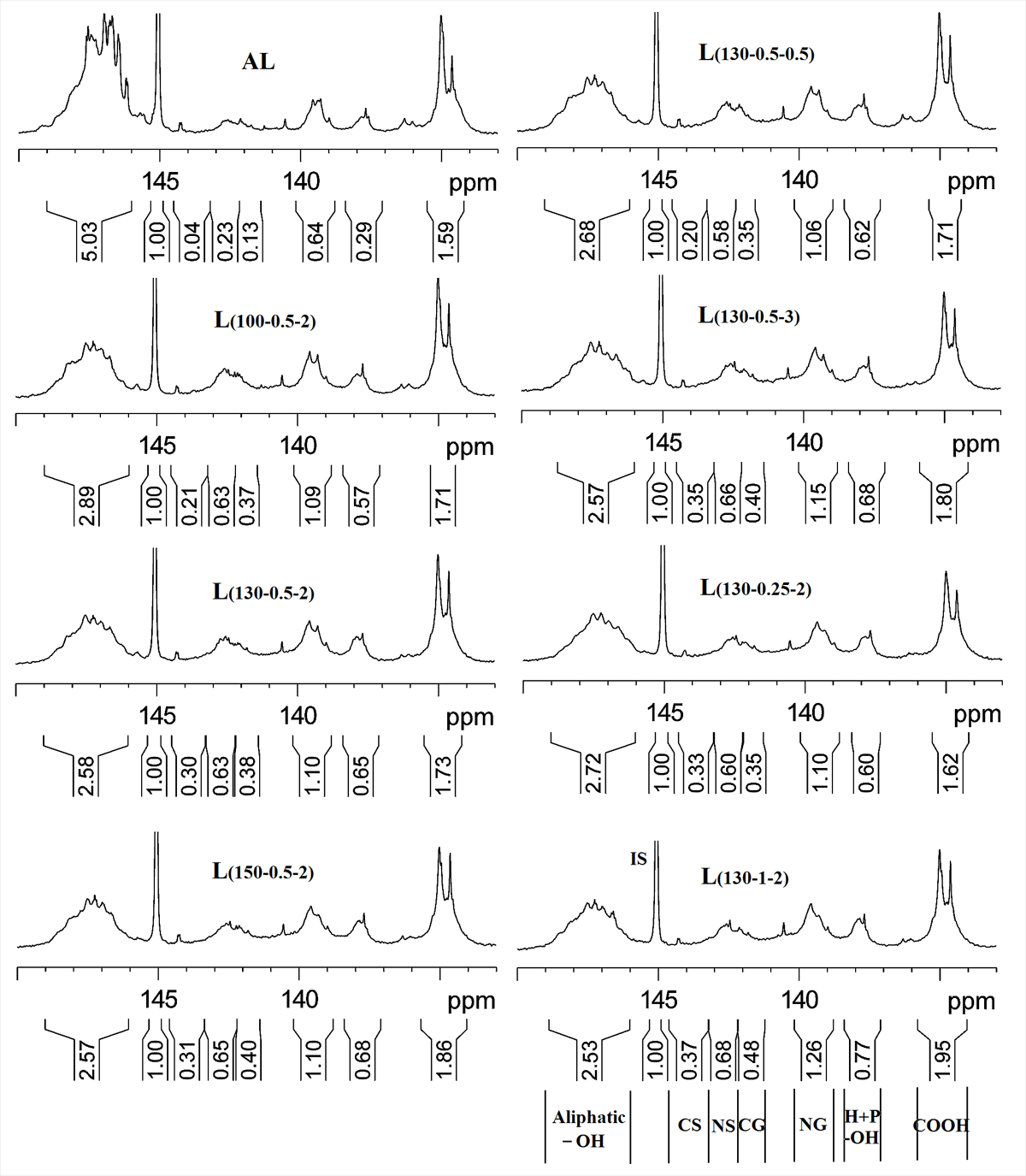

Supplement: Supplementary file 1 — Additional file 1: Table S1. The byproducts liberated during the dilute acid pretreatment. Table S2. Yield and contents of associated polysaccharides (absolute %, w/w) of the lignins isolated with 1.5 % NaOH at 80 °C for 2 h from the dilute acid pretreated rice straw. Table S3. Weight-average (Μ w) and number-average (Μ n) molecular weights, and polydispersity (Μ w /Μ n) of the alkaline lignin fractions. Table S4. Assignments of 13C-1H cross-peaks in HSQC spectra of the alkaline lignins recovered from the integrated process. Figure S1. FT-IR spectra of the raw material, acid pretreated straw and alkali post-treated straw. Figure S2. SEM images of the raw material, acid pretreated straw and alkali post-treated straw. Figure S3. XRD patterns of the raw material, acid pretreated straw and alkali post-treated straw. Figure S4. 31P-NMR spectra of the lignins obtained from the integrated process under various processing conditions. [file 13068_2016_632_MOESM1_ESM.doc]
